# Supplementary material for: Safety and Effectiveness of Vedolizumab and Ustekinumab in Elderly Patients with Inflammatory Bowel Disease: A Real-Life Multicentric Cohort Study
Source: J Clin Med. 2024 Jan 9;13(2):365. doi: 10.3390/jcm13020365 (PMC10816258; doi:10.3390/jcm13020365)
Supplement: Supplementary file 1 [file jcm-13-00365-s001.zip › jcm-2766000-supplementary.pdf]

Supplementary Tables

Supplementary Tables S1. Corticosteroid free remission at 6 months in the elderly cohort treated with vedolizumab or ustekinumab.

	Exp(B)	95% CI interval	P value
Studied biological (UST)	0.449	0.200-1.007	0.052
IBD type (CD)	0.712	0.050-10.104	0.802
Previous anti-TNF	0.664	0.153-2.879	0.584
Previous surgery	0.619	0.272-1.407	0.252
Propensity score elderly	2.161	0.004-1305.49	0.814
Constant	1.416		0.304

Supplementary Table S2. Corticosteroid free remission at 1 year in the elderly cohort treated with vedolizumab or ustekinumab.

	Exp(B)	95% CI interval	P value
Studied biological (UST)	0.432	0.184-1.011	0.053
IBD type (CD)	3.018	0.187-48.769	0.436
Previous anti-TNF	1.311	0.285-6.030	0.728
Previous surgery	0.779	0.340-1.787	0.556
Propensity score elderly	0.231	0.000-192.36	0.669
Constant	1.058		0.875

Supplementary Table S3. Outcomes in elderly UC versus CD patients

	**UC (n=70)**	**CD (n=101)**	**p-value**
**Corticosteroid-free remission (yes)**			
**Week 8-14**	20/68 (29.4%)	17/96 (17.7%)	0.077
6 months	33/64 (51.6%)	35/90 (38.9%)	0.119
1 year	29/59 (49.2%)	42/82 (51.2%)	0.809
**2 year**	25/44 (56.8%)	30/65 (46.2%)	0.275
**Endoscopic remission (yes)**			
**6 months**	5/15 (33.3%)	3/15 (20%)	
**1 year**	11/21 (52.4%)	12/27 (44.4%)	
**Safety**			
**Any AE (**yes)	28/70 (40%)	41/101 (40.6%)	0.938
Any infection (yes)	10/70 (14.3%)	11/101 (10.9%)	0.506
Gastro-intestinal infection	3/70 (4.3%)	3/101 (3%)	0.690
**Any serious adverse event (yes)**	13/70 (18.6%)	29/101 (28.7%)	0.130

**Hospitalisation for IBD flare**	7/70 (10%)	12/101 (11.9%)	0.700
Hospitalisation (not for IBD flare)	4/70 (5.7%)	14/101 (13.9%)	0.088
Malignancy	1/70 (1.4%)	3/101 (3%)	0.645
**Death**	0	2/101 (2%)	0.514
